# The Roles of Non-Coding RNAs in Tumor-Associated Lymphangiogenesis

**DOI:** 10.3390/cancers12113290

**Published:** 2020-11-06

**Authors:** Khairunnisa’ Md Yusof, Rozita Rosli, Maha Abdullah, Kelly A. Avery-Kiejda

**Affiliations:** 1 Department of Biomedical Sciences, Faculty of Medicine and Health Sciences, Universiti Putra Malaysia, Selangor 43400, Malaysia; KhairunnisaBintiMdYusof@uon.edu.au (K.M.Y.); rozita@upm.edu.my (R.R.); 2 Priority Research Centre for Cancer Research, Innovation and Translation, School of Biomedical Sciences and Pharmacy, Faculty of Health and Medicine, University of Newcastle, Newcastle, NSW 2308, Australia; 3 Medical Genetics, Hunter Medical Research Institute, New Lambton Heights, NSW 2305, Australia; 4 Department of Pathology, Faculty of Medicine and Health Sciences, Universiti Putra Malaysia, Selangor 43400, Malaysia; maha@upm.edu.my

**Keywords:** ncRNA, miRNA, lncRNA, LEC, tumor-associated lymphangiogenesis, lymphatic

## Abstract

**Simple Summary:**

The lymphatic system plays key roles in the bodies’ defence against disease, including cancer. The expansion of this system is termed lymphangiogenesis and it is orchestrated by factors and conditions within the microenvironment. One approach to prevent cancer progression is by interfering with these microenvironment factors that promote this process and that facilitate the spread of cancer cells to distant organs. One of these factors are non-coding RNAs. This review will summarize recent findings of the distinct roles played by non-coding RNAs in the lymphatic system within normal tissues and tumours. Understanding the mechanisms involved in this process can provide new avenues for therapeutic intervention for inhibiting the spread of cancer.

**Abstract:**

Lymphatic vessels are regarded as the ”forgotten” circulation. Despite this, growing evidence has shown significant roles for the lymphatic circulation in normal and pathological conditions in humans, including cancers. The dissemination of tumor cells to other organs is often mediated by lymphatic vessels that serve as a conduit and is often referred to as tumor-associated lymphangiogenesis. Some of the most well-studied lymphangiogenic factors that govern tumor lymphangiogenesis are the vascular endothelial growth factor (VEGF-C/D and VEGFR-2/3), neuroplilin-2 (NRP2), fibroblast growth factor (FGF), and hepatocyte growth factor (HGF), to name a few. However, recent findings have illustrated that non-coding RNAs are significantly involved in regulating gene expression in most biological processes, including lymphangiogenesis. In this review, we focus on the regulation of growth factors and non-coding RNAs (ncRNAs) in the lymphatic development in normal and cancer physiology. Then, we discuss the lymphangiogenic factors that necessitate tumor-associated lymphangiogenesis, with regards to ncRNAs in various types of cancer. Understanding the different roles of ncRNAs in regulating lymphatic vasculature in normal and cancer conditions may pave the way towards the development of ncRNA-based anti-lymphangiogenic therapy.

## 1. Introduction

The lymphatic system is often regarded as the “forgotten” circulatory system due to limited resources and the fact that it is less well-studied. However, findings on novel specific markers in the development of lymphatic endothelial cells (LECs) during embryogenesis, such as the vascular endothelial growth factor (VEGF-C), lymphatic vascular endothelial cell hyaluronin receptor 1 (LYVE-1), podoplanin, and homeobox transcription factor 1 (PROX-1), have led to their extensive investigation and an understanding of their mechanism of action in lymphangiogenesis, in normal and pathological conditions, such as cancer. Tumor growth is strictly dependent on the cross talk and interaction between tumor cells and the tumor microenvironment that induces genomic instability and alters the surrounding tissue to promote tumor spread through lymphatic channels [1,2]. Previous findings suggest that the higher permeability and discontinuous structure of the lymphatic vessels (which contain many open junctions and pores), accompanied by the minimal shear stress of lymph flow, provide an ideal environment for the dissemination of tumor cells via lymphatic vessels when compared to the bloodstream [3,4].

With the advancement of technologies in bioinformatics, deep and genome-wide sequencing has revealed new players that are involved in the development of lymphangiogenesis, including non-coding RNAs (ncRNAs) [5]. These ncRNAs are non-coding transcripts that are not translated into protein, but play crucial roles in regulating gene expression in normal physiology and biological processes. Accordingly, their dysregulation can affect subsequent genomic regulation, leading to disease development. In this review, we aim to discuss the roles of ncRNAs, mainly microRNAs (miRNAs) and long non-coding RNAs (lncRNAs), in lymphatic development and tumor-associated lymphangiogenesis.

## 2. Overview of the Lymphatic System

### 2.1. Structure and Functions of Lymphatic Vasculature

**Figure 1:**
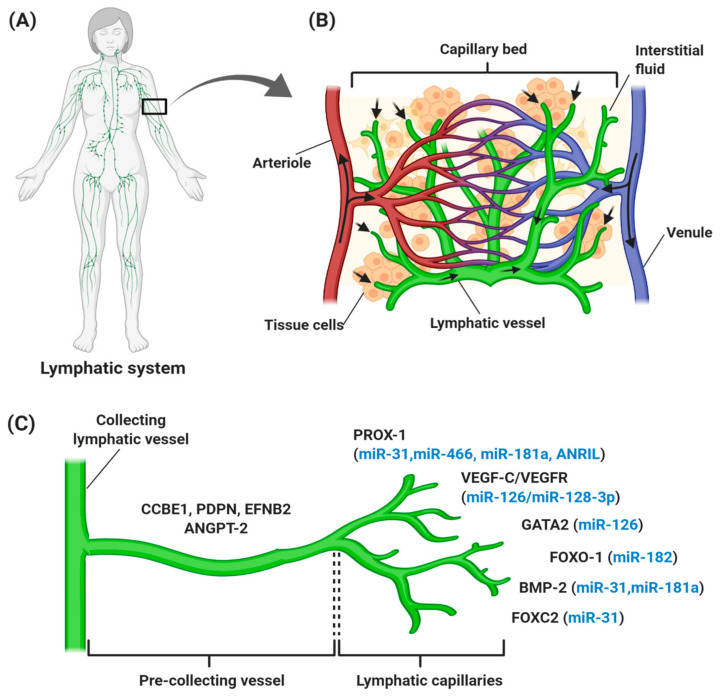
Overview of the human lymphatic structure and developmental lymphangiogenesis. (**A**) An anterior view of the lymphatic system with lymph nodes and vessels. (**B**) Structural relationship of the lymphatic system with the circulatory system, which consists of a capillary bed containing lymphatic vessels intertwined between blood vessels. Blood enters the arteriole (red), is filtered through blood capillaries, and moves into tissue spaces. While most of the interstitial fluid moves back into the venule (blue), some of them drain into lymphatic capillaries to become lymph and proceed to lymph nodes. (**C**) Lymphangiogenesis under physiological conditions with specific genes and lymphangiogenic factors that regulate the process. Vascular endothelial growth factor receptor (VEGFR-3) becomes more restricted to lymphatic endothelial cell (LEC) precursors and starts to express neuropilin-2 (NRP-2), increasing the sensitization of LEC towards VEGF-C, which subsequently stimulates VEGF-C/VEGFR-3 signaling to induce the lateral budding of LEC and lymphatic sprouting. VEGF-C/VEGFR-3 signaling continuously upregulates additional LEC markers, such as forkhead box 1 and C2 (FOXO1 and FOXC2), podoplanin (PDPN), angiopoietin-2 (ANGPT-2), ephrin B2 (EFNB2), bone morphogenesis protein-2 (BMP-2), and GATA-binding protein 2 (GATA2), which are involved in developmental lymphangiogenesis, including vessel sprouting, lymphatic maturation, and the remodeling of lymphatic vasculature. The lymphangiogenic factors expressed throughout the process are also regulated by different ncRNAs (blue). It is common for miRNAs to regulate more than one gene, thereby explaining the expression of miR-126, miR-181a, and miR-31 at different stages of lymphatic development.

The lymphatic vascular system is specialized in transporting solutes, macromolecules, and antigens from tissues back into the blood circulation in the form of lymph, and thus plays a central role in maintaining homeostasis in mammals. The lymphatic system also serves as a conduit for immune cells, such as antigen-presenting dendritic cells, macrophages, and memory T lymphocytes, from peripheral tissues to lymph nodes and other lymphoid organs, such as the spleen, tonsils, and thymus [6,7,8]. Generally, lymph from the right upper arm, thorax, and head is returned via the right lymphatic trunk to connect with the right subclavian vein, while lymph from the left side is collected from lower limbs, the abdomen, and the thoracic duct to the left subclavian vein [9]. These collecting lymphatic vessels are connected with an abundance of lymph nodes that filter the pathogens (Figure 1A). An average of 8–12 L lymph fluid with 20–30 g/L of protein fluid accumulates in the tissue and is transported through the lymphatic system daily in humans [10,11]. In contrast with the blood vasculature system, the uniquely structured lymphatic system is unidirectional (fluid only moves from tissue to the circulatory system), consisting of blind-ended and thin-walled vessels, composed of LECs, with no pericytes or smooth muscle cells encompassing the vessels. However, lymphatic capillaries maintain their inter-junctional gaps by “button-like” junctions that are composed of vascular endothelial cadherin (VE-cadherin), and act as a leukocyte entry point to the vessels, thereby rendering high permeability of the wall and optimal uptake of lymph components [6,12,13]. As depicted in Figure 1B, small lymph capillaries interact with blood capillaries in the interstitial space in tissues. Under physiological conditions, arterial blood is consistently filtered through semi-permeable blood capillaries, where blood plasma enters extracellular tissue spaces. The differences in pressure between interstitial fluid and lymphatic vessels permit the fluid to enter the lumen. As fluid enters the lumen, the intraluminal pressure decreases and the junctions begin to close, thereby preventing the backflow of fluid into the interstitium [7,14,15].

### 2.2. Regulators in Developmental Lymphangiogenesis

Generally, lymphatic development takes place during embryogenesis, but does not occur in a healthy adult, except in the ovaries and mammary tissue during the female reproductive cycle. The earliest definitive sign of lymphatic development is the presence of LYVE-1 in the embryonic cardinal veins, which permits the tissue to become “competent” to respond to specific lymphatic-inducing signals [16,17]. Upon its polarized induction, the transcription factor PROX-1, which is a master regulator of LECs, starts to be expressed in the cardinal vein [17,18]. PROX-1 is activated upon the interaction of SRY-related HMG-box 18 (SOX18) with the transcription factor chicken ovalbumin upstream promoter-transcription factor II (COUP-TFII), which is essential for venous endothelial cell identity and the upregulation of LEC-specific genes, such as vascular endothelial receptor 3, VEGFR3, and neuroplilin-2 (NRP2) [19,20]. LECs with PROX-1 expression then sprout to form primary lymphatic plexus and sacs, and migrate away from the cardinal and intersomitic veins [21,22]. The final steps of lymphatic development include differentiation into collecting lymphatic vessels and lymphatic capillaries that are driven by a complex range of signaling events, such as VEGFR-3/VEGF-C and calcium-binding epidermal growth factor domain-containing protein 1 (CCBE-1) signaling pathways, together with proteins NRP-2,ephrin B2 (EFNB2) [23,24], PROX-1, LYVE-1, and angiopoietins (ANGPTs) [25].

Lymphangiogenesis refers to the growth of lymphatic vessels by the sprouting of new vessels from pre-existing lymphatic vessels (Figure 1C). It has been reported that lymphangiogenesis is governed by VEGF-C/VEGF-D/VEGF-3 signaling. VEGF-C, together with VEGF-D, binds and activates the tyrosine kinase receptors, and VEGFR-3 and the co-receptor NRP-2 on LEC, thereby promoting the migration of LECs for venous sprouting [24,26]. Meanwhile, CCBE-1 is a secreted protein expressed in tissues related to budding-venous-derived LECs and is predicted to bind with extracellular matrix components to induce the effect of VEGF-C in vivo [27]. In the later stage of lymphatic development, the further expansion of lymphatic vasculature involves vessel sprouting, lymphatic valve formation, platelet aggregation, and remodeling to form the lymphatic vascular tree [28,29]. During lymphatic valve formation, forkhead box C2 (FOXC2) transcription factor, PROX-1, and globin transcription factor (GATA) expression are upregulated in cells [30]. PROX-1 controls the interaction of pericytes with LECs [31], while its regulation with FOXC2 controls the expression of the gap junction protein connexin 37, and activation of nuclear factor activated-T cell 1 (NFATc1) signaling [32,33]. Also, PROX-1 regulates cell surface molecule members; such as the cadherin EFG LAG seven pass type G-receptor (CELSR1), Vang-like 2 (VANGL2), Notch and bone morphogenetic protein (BMP) [33,34,35]. Platelet aggregation is important in separating the blood and lymphatic vessel compartments and this process is controlled by the presence of podoplanin on the surface of LECs, to bind with platelet C-type lectin like receptor2 (CLEC-2), thereby preventing blood from entering the lymphatic vessels [28,36]. During the later stage, signaling pathways like ANGPT and EFNB2 are activated during the remodeling of the primitive lymphatic plexus for pre-collecting and collecting vessels [37,38].

## 3. Non-Coding RNAs

**Figure 2:**
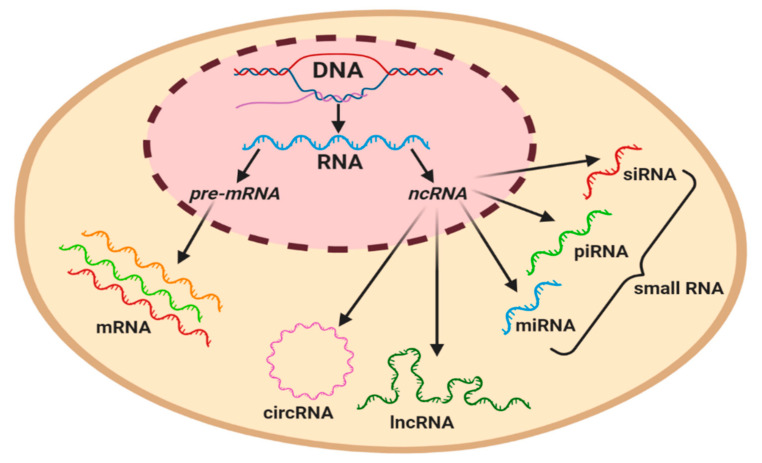
Graphical classification of coding and regulatory non-coding RNAs in a eukaryotic cell. Precursor mRNA (pre-mRNA) gives rise to mRNA. ncRNAs can be subcategorized based on their length, with the longest circulating RNA (circRNA) and long non-coding RNAs (lncRNAs) being ~200 nt in length, whilst miRNA (19–23 nt), piwi-interacting RNA (piRNA) (23–32 nt), and small interference RNA (siRNA) (20–25 nt) are classified as small RNAs.

The majority of studies conducted on the human genome have reported that only 2% of transcripts are coded and translated into proteins. The remaining transcripts are considered as non-coding RNAs, representing a cluster of functional molecules that do not translate into proteins, but play a pivotal role in the regulation of gene expression [39]. Numerous studies have classified regulatory ncRNAs based on the length of nucleotides, of which long non-coding RNAs (lncRNAs) usually have more than 200 nucleotides, while an RNA molecule with less than 200 nucleotides is classed as a short ncRNA, such as microRNAs (miRNAs), circulating RNAs (circRNA), piwi-interacting RNAs (piRNAs), and small interference RNA (siRNA) (Figure 2) [39,40]. ncRNAs are involved in numerous biological processes, including the regulation of genes, proteins, chromosome structures, cell development, proliferation, and survival. In this section, the synthesis, classification, and functions of ncRNAs with special reference to miRNAs and lncRNAs will be discussed, as well as the mechanisms involved in regulating lymphatic development under physiological conditions.

### 3.1. Synthesis, Classification, and Functions of ncRNAs

The most studied class of ncRNAs is miRNAs, which are relatively small ncRNAs with an average length of ~22 nt that are generally involved in gene silencing by controlling the translation of mRNA. miRNAs regulate the expression of genes involved in many biological processes, including proliferation, differentiation, apoptosis, and cell development [41,42,43]. The majority of miRNAs are transcribed from intergenic regions, whilst others are from exonic and intronic regions of the human genome. The synthesis of miRNAs takes place through a multi-step process, starting with the transcription of primary miRNAs (pri-miRNAs) by RNA polymerase II, before being processed into precursor miRNAs (pre-miRNAs) by RNAse III enzymes. [44,45]. Next, the pre-miRNAs are exported into the cytoplasm and cleaved by the RNase Dicer-TAR RNA-binding protein (TRBP) complex and eventually produced mature, single strand miRNAs with a length of 19–23 nt [46,47]. miRNAs regulate specific genes by guiding a diverse set of multi-protein RNA-induced silencing complexes (RISCs), which are comprised of the argonaut (Ago) and glycin-tryptophan (GW) families [48]. The loading of miRNAs into RISCs directs the regulation of mRNA by recognizing a complementary sequence of mRNA that is located at the 3′-UTR, and eventually, the expression of mRNA is repressed via two mechanisms; the degradation of mRNA and inhibition of mRNA translation [49,50,51]. The discovery of the function of ncRNAs has led to them being extensively studied in diseases, including cancer. The dysregulation of ncRNAs has been reported to play roles in the carcinogenic process, resulting from multiple mechanisms at the transcriptional or post-transcriptional level. For instance, DNA hypermethylation of the promoter miRNA leads to miRNA silencing at the transcriptional level [52].

LncRNAs are a heterogenous group of ncRNA of ~200 nt in length, with more than 120,000 transcripts reported to be encoded in the human genome [53,54]. In eukaryotic genomes, lncRNAs are transcribed from different DNA elements, such as enhancers, promoters, and intergenic regions [55]. The majority of lncRNAs are synthesized by RNA polymerase II and spliced, polyadenylated, and 5′-capped, but some of them are not adenylated. Moreover, the synthesis of lncRNAs is under the control of cell type and stage-specific stimuli and involves multiple mechanisms, including cleavage by ribonuclease P (RNaseP) to generate mature ends, the formation of small nucleolar (snoRNA) and protein caps at their ends, and the formation of circular structures [56,57]. LncRNAs are basically classified as the following based on their genomic loci and function: (1) Sense lncRNAs–synthesized from exons of protein genes utilizing the same promoter region of the gene; (2) antisense lncRNAs–synthesized from the opposite strand of the protein coding region; (3) intronic lncRNAs–generated from an intron of a protein coding region; (4) enhancer lncRNAs– synthesized from transcription factor binding regions; (5) intergenic lncRNAs–encoded between protein coding genes and transcribed independently; (6) circular forms–3′ and 5′ ends are covalently enclosed to create a circular loop derived from the splicing of a protein coding gene; and (7) bi-directional forms–transcribed from the same promoter as coding genes, but in the opposite direction [58,59,60].

Unlike miRNAs, lncRNAs interact with other molecules, such as RNA, DNA, and proteins in exerting the functions of normal cells [61,62]. LncRNAs play roles through multiple mechanisms to regulate chromatin modeling, protein functionality and gene expression, as well as the translational activity and decay of mRNAs [63,64]. Interestingly, some lncRNAs have been reported to harbor miRNAs targeting sites and can act as endogenous miRNA sponges to suppress the inhibitory effects of miRNAs on mRNA translation and stability [54]. Due to their multiple roles in various biological functions, the deregulation or aberrant expression of lncRNAs can lead to pathological conditions, including cancer [54,63,64]. The mechanisms by which lncRNAs contribute to cancer development are diverse, but are mainly involved in fundamental processes such as self-renewal, cell cycle regulation, the DNA damage response, proliferation, apoptosis, and metastasis [65].

Another class of ncRNAs is piRNA, named after the PIWI protein and this class is 23–32 nt in length. Mature piRNAs are derived from two major steps: (1) Precursors transcribed from piRNA clusters and cleavage by the PIWI protein and (2) modification through a ‘ping-pong’ cycle. Distinct from miRNA and siRNAs, piRNAs are processed in a Dicer-independent mechanism which only functions through binding with PIWI proteins and as a defense against transposons in germ cells [66,67]. Small interfering RNA (siRNA) is a double stranded RNA with a 20–25 nt length that plays essential roles in RNA interference RNA (RNAi). siRNAs can be divided into two types: Exogenous siRNA (produced from exogenous nucleic acid resulting from artificial insertion or viral infections) and endogenous siRNAs (which arise from endogenous genomic loci and are transcribed from transposon elements (TEs)) [68,69].

### 3.2. Regulation of Developmental Lymphangiogenesis by Non-Coding RNAs

As mentioned earlier, ncRNAs mainly miRNAs and lncRNAs, play significant roles in regulating gene expression. A number of studies have distinguished different roles for miRNAs in regulating genes associated with various biological processes such as angiogenesis [70,71], modulation of the immune system [72], and oxidative stress [73,74]. The profiling of ncRNAs in LECs led to the discovery that lymphatic development can also be regulated by miRNAs (Figure 1C).

An example of a regulatory miRNA in lymphangiogenesis is miR-31, which acts as a negative regulator of LEC signature genes. A gain-of-function experiment of miR-31 in *Xenopus* and zebrafish embryos resulted in extensive disruption of lymphangiogenesis and lymphatic vessel development. These findings were supported by the overexpression of miR-31 in LEC, that preferentially repressed LEC signature genes [75]. miR-31 was found to mediate the repression of FOXC2, a transcription factor that is required for the specification of collecting lymphatic vessels and lymphatic capillaries as well as receptor activity modifying protein-2 (RAMP2) that induces lymphangiogenesis through adrenomedullin signaling [76,77,78]. In contrast, BMP2, a negative modulator in lymphatic fate development, was shown to induce miR-31 and miR-181a expression in a Smad4-dependent manner [79]. On the other hand, miR-181a negatively regulated PROX-1 mRNA expression. A substantial decrease in PROX-1 expression during lymphatic development in an adult is associated with a significant increase of the miR-181a level, thereby confirming its contribution to the temporal control of PROX-1 levels in maintaining LEC identity [80]. 

More recent studies have identified the role of miR-126 in governing lymphatic development in both in vitro and in vivo models. miR-126 was shown to specifically regulate lymphatic development, possibly by modulating fms-related tyrosine kinase-4 (FLT4) (or VEGFR) signaling without affecting PROX-1 cells in the cardinal vein [81]. The findings are in line with a study by Chen et al. which demonstrated that miR-126 regulates LEC sprouting and proliferation through CXCL12a-mediated chemokine signaling, and that it may be involved in the lymphatic pathogenesis of cardiovascular diseases [82]. Another target gene of miR-126 is GATA2, which regulates lymphatic valve development and the maturation of lymphatic vasculature in mammals. Both GATA2 and mir-126 are required for the expression of cell junction molecules, such as claudin-5 and VE-cadherin. Interestingly, the overexpression of mir-126 could rescue junction defects in human LECs lacking GATA2 [83].

An anti-lymphangiogenic factor, miR-128-3p was shown to be a negative regulator for VEGF-C. miR-128-3p was reported to target and inhibit VEGF-C through calcium (Ca2+) signaling and subsequently suppressed LEC proliferation [84]. Another study by Seo et al. reported that a high expression of miR-466 targeted a 7mer-1A site in the 3′ UTR of PROX-1 to inhibit the expression of PROX-1 at mRNA and protein levels in the burn corneal injury model. Considering that PROX-1 is important in regulating VEGF-C and ANGPT-2, its downregulation by miR-466 therefore suppressed lymphangiogenesis and angiogenesis in vivo [85]. A study in a zebrafish model by Kiewsow and co-workers demonstrated miR-182 as a novel target for JunB, a subunit of the transcription factor AP-1, which plays essential roles in vasculature development. The downregulation of JunB and miR-182 resulted in a complete failure in lymphatic vessel formation. Additionally, regulation of the JunB/miR-182 axis was shown to control precise FOXO1 levels that are required for proper lymphatic vasculature development in zebrafish [86].

An increasing amount of evidence shows that lncRNAs play pivotal roles in cell development and differentiation, post-transcriptional regulation, and tumorigenesis [87,88]. Despite limited studies on lncRNAs in lymphatic development, antisense noncoding RNA in the INK4 locus or ANRIL has been reported to influence lymphangiogenesis that involves wound healing in diabetes, by regulating miR-181a/PROX-1 signaling. Mechanistically, miR-181a repressed PROX-1 translation by directly binding to the 3′-UTR region, while ANRIL sponged the inhibitory effect of miR-181a. The overexpression of ANRIL and inhibition of miR-181a suppressed apoptosis via a caspase-dependent pathway which subsequently promoted lymphatic vessel formation [89,90]. In a study on coronary heart disease, ANRIL (DQ485454) was demonstrated to regulate endothelial cell (EC) functions, including monocyte adhesion to ECs, transendothelial migration of monocytes and EC migration by regulating LYVE-1, as well as tube formation and migration transcription factors CLAP-GLY linker containing protein 1 (CLIP1) and ezrin (EZR) [91].

## 4. Tumor-Associated Lymphangiogenesis

**Figure 3:**
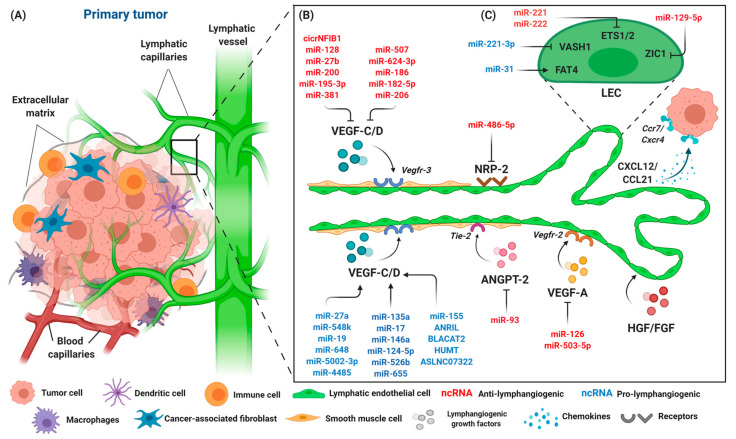
The components of the primary tumor microenvironment and lymphangiogenic factors involved in tumor progression. (**A**) Tumor cells are surrounded by an abundance of cell types, such as immune cells, macrophages, dendritic cells, fibroblasts, and endothelial cells. Blood and lymphatic capillaries are required to support the growth of the tumor, while serving as a conduit for the dissemination of tumor cells to distant organs. (**B**) Lymphangiogenesis occurs during tumor progression with lymphangiogenic factors released by tumor cells, namely VEGF-C/D, VEGF-A, NRP-2, ANGPT-2, and HGH/FGF. Tumor cells that expressed chemokine receptors CXCR4 are attracted to inflammatory chemokines CCL21/CXCL21 released by LECs, and thus stimulate lymphangiogenesis. The interaction of LECs with lymphangiogenic factors results in an increase of lymphatic permeability by changing the adhesion properties of LECs, accompanied by the dilation of collecting lymphatic vessels to facilitate the spread of tumor cells through intratumoral and peritumoral lymphangiogenesis, finally leading to lymphatic metastasis. A number of different ncRNAs are expressed throughout the process. ncRNAs exert their pro- (blue) or anti-lymphangiogenic (red) effect by regulating primary lymphangiogenic components. (**C**) Regulation of tumor-associated lymphangiogenesis by ncRNAs also occurs on LEC transcription factors, such as protocadherin Fat4 (FAT4), vasohibin-1 (VASH1), Ets proto-oncogene 1 and 2 (ETS1 and ETS2), and Zinc finger protein 1 (ZIC1). Keywords: Vascular endothelial growth factor (VEGF-C and VEGF-A); vascular endothelial growth factor receptor (VEGFR-3/2); neuropilin-2 (NRP-2); angiopoietin-2 (ANGPT-2); fibroblast growth factor (FGF); hepatocyte growth factor (HGF); C-C chemokine receptor (CCR); C-C chemokine ligand (CCL); and C-X-C motif chemokine (CXCL).

Lymphangiogenesis can be induced in certain pathological conditions, such as chronic inflammation, tumors, and wound healing [92,93]. The lymphangiogenesis that occurs in tumors involves other types of lymphatic remodeling and modulation of immune functions that serve as a conduit to tumor cells to increase metastatic spread to lymph nodes and distant organs. Tumor cells invade the microenvironment after the tumor reaches a diameter of 1 mm and disrupt the newly formed blood vessels, enabling the passive entry of tumor cells where they can then metastasize (Figure 3A). As the metastasis involves both blood and lymphatic circulation, it has been shown that tumor cells detach from their primary sites before entering tumor-associated absorbing lymphatic (TAAL) vessels through intra-endothelial channels, allowing the cells to enter the lymphatic circulation [94,95].

### 4.1. Genes Involved in Tumor-Associated Lymphangiogenesis

Lymphatic remodeling occurs when the tumor microenvironment stimulates tumor cells, tumor stroma cells, and tumor-infiltrating cells to induce a series of lymphangiogenic factors. It starts during primary tumor lymphangiogenesis, where LECs play an active role in the interactions of tumor cells with lymphatic vessels. The morphological changes that occur when LECs become hyperplastic are induced directly by tumor-secreted VEGF-C, VEGF-D, and ANGPT, resulting in increased contact between LECs and tumor cells [96,97], thus increasing vascular permeability and changing the adhesive properties of the LECs [25]. Meanwhile, other growth factors, including fibroblast growth factors and inflammatory cytokines, are regulated by VEGF-C expression levels, further suggesting the involvement of VEGF-C in lymphatic development and lymphangiogenesis [97]. Both VEGF-D and VEGF-C require proteolytic maturation mediated by CCBE1 and a disintegrin and metalloprotease domains with thrombospondin (ADAMTS3), before reaching the maximal affinity for VEGFR-3 and its co-receptor NRP-2 to exert lymphangiogenic activity [98]. Unlike VEGF-D, VEGF-C induces cyclooxygenase-2 (COX-2), an enzyme involved in the synthesis of prostaglandin expression in endothelial cells, hence promoting the dilation of collecting lymphatic vessels and facilitating the spread of tumor cells through intratumoral and peritumoral lymphangiogenesis [99]. In contrast, VEGF-D was found to regulate the enzyme that breaks down prostaglandin in endothelial cells, namely 15-hydroxyprostaglandin dehydrogenase (15-PGDH), resulting in the prolonged exposure of LECs in collecting lymphatics to prostaglandins and enlargement of the lymphatic vessels, hence allowing the dissemination of tumor cells [100,101]. The increased level of VEGF-C was found to have accelerated the growth of tumors in renal cell carcinoma [102],chondrosarcoma [103,104], breast [105,106,107], colon [108], lung [109], and skin cancer [110,111]. Of note, the prostaglandin-lymphangiogenesis induction is not specific to cancer, but also occurs in the inflammation setting.

VEGF-A exerts its effect by binding to VEGFR-2 that is also present on LECs and indirectly induces LEC proliferation and lymph node metastasis in mouse tumor xenograft models [112]. ANGPT, on the other hand, promotes peritumoral lymphangiogenesis, as demonstrated by the transgenic expression of both ANGPT-1 and ANGPT-2 in pancreatic B cells of the Rip1Tag2 experimental mouse model [113]. Unlike ANGPT-1, ANGPT-2 plays a crucial role with VEGF-A in tumor lymphangiogenesis. VEGF-A was shown to increase the expression of ANGPT-2 in endothelial cells via the NFAT pathway, thereby leading to tumor lymphangiogenesis and lymph node metastasis in mice with pancreatic and lung tumors [114,115]. Furthermore, the treatment of ANGPT2-blocking antibodies was shown to suppress tumor-associated lymphangiogenesis and promote endothelial cell–cell junctions [115]. Additionally, HGF and FGF2 have been shown to be increased during lymphangiogenesis. The findings were correlated with a highly expressed cognate receptor of HGF/c-Met during inflammation and in cancer-associated lymphatic vessels in both in vitro and in vivo studies, suggesting a direct role of HGF with LECs [116,117,118]. Fibroblast growth factor 2 (FGF2) can synergize with VEGF-C signaling and induce lymphatic vessel formation and tumor metastasis [119,120]. Inflammatory cytokines may play important roles in inducing lymphangiogenesis in tumors as cancer progression is often accompanied by inflammation. Cytokines, including interleukin-1β (IL-1β), IL-6, tumor necrosis factor-alpha (TNF-α), and chemokines were found to be highly expressed in tumor-associated inflammation. IL-1β and TNF-α induce VEGF-C expression in tumor-associated macrophages, hence promoting lymphangiogenesis and lymph node metastasis [121,122]. Besides inducing new lymphatic vessels, tumor cells possess the ability to incorporate the existing lymphatic system at the primary sites. The chemokine receptors CCR7 and CXCR4, which are expressed by invading cancer cells, bind to secreting chemokines such as CCL21 and CXCL12, to recruit more cancer cells towards lymphatic vessels [123,124]. A summary of genes involved in lymphangiogenesis in tumors is shown in Figure 3B.

### 4.2. Regulation of Tumor-Associated Lymphangiogenesis by Non-Coding RNAs

Emerging studies have implicated the connection between lymphatic development and lymphangiogenesis with ncRNAs under normal physiological conditions. These regulatory RNAs also play significant roles in pathological conditions by altering gene and protein expression, interacting with RNA molecules, or acting as scaffolds for protein complexes to redirect signaling pathways [125,126]. Considering that lymphatic metastasis is facilitated by tumor lymphangiogenesis that serves as a conduit for tumor cells to disseminate into distant organs, targeting the precursors or factors including the regulatory ncRNAs related to the process provides a possibility to assist in new therapeutic development for lymphangiogenesis-related diseases. Different methods and techniques have been employed to quantitate the level of ncRNAs both absolute and relative quantification, including quantitative real-time PCR, microarrays, and immunoblotting. However, the outcomes of the studies vary, based on the objective and the assays applied. For instance, some studies have reported and confirmed miRNA binding to 3′-UTR on target genes directly, while others have only reported on expression levels and the correlation between ncRNAs and the induction of lymphangiogenesis in the tumor microenvironment, by analyzing different tissues (summarized in Table 1). In this section, we discuss the regulatory roles of ncRNAs, with special reference to miRNAs and lncRNAs, in tumor-associated lymphangiogenesis in various types of cancer (Figure 3B,C).

#### 4.2.1. MiRNAs in Tumor-Associated Lymphangiogenesis

Studies have demonstrated that miRNAs play an important role in lymphangiogenesis in various types of cancer, with several VEGF proteins being targeted (refer to Table 1 for specific proteins and mechanisms). An anti-lymphangiogenic miRNA, miR-128 directly targets VEGF-C, by reducing both angiogenesis and lymphangiogenesis in cell culture and xenograft models. The high expression of miR-128 resulted in the suppression of non-small cell lung carcinoma (NSCLC) cell proliferation, inhibition of colony formation, and induction of cell cycle arrest during the G1 phase. Furthermore, restoration of miR-128 was shown to inhibit tumorigenicity of A549 cells in nude mice by directly targeting VEGF-C and subsequently downregulating the phosphorylation of ERK, AKT, and p38 [127]. These findings are in line with a study on miR-128 downregulation that inhibited the proliferation of T24 and 5637 bladder cancer cells by targeting VEGF-C [128]. A member of the miR-23~27 cluster, miR-27 was found to be highly expressed in endothelial cells, and the downregulation of miR-27b significantly inhibited the tube formation of blood endothelial cells in vitro [46]. While the finding suggested pro-angiogenic properties of miR-27, more recent studies have reported that miR-27a/b plays distinct roles in tumor lymphangiogenesis, through its regulator VEGF-C. In human chondrosarcoma, adipocyte secretions such as leptin and adiponectin regulate the expression of VEGF-C by downregulating miR-27b, particularly in different pathways. The downregulation of miR-27b by leptin occurred through FAK, PI3K, and Akt pathways, while CaMKII, AMPK, and p38 signaling was regulated by adiponectin. Subsequently, the activation of VEGF-C promotes the lymphangiogenesis of LECs in the chondrosarcoma microenvironment [104,129].

**Table 1:** Summary of ncRNAs that mediate lymphangiogenesis and their targets in different tumor microenvironments, with the specific mechanisms, types of tissues, and discovery platforms applied in reported studies.

ncRNA Expression	Targets	Disease Model	Mechanism	Type of Tissue	Discovery Platform	Ref.
**Anti-lymphangiogenic**						
↓miR-27b	↑leptin, ↑VEGF-C, ↑FAK, ↑PI3K/Akt signaling	Chondrosarcoma	Correlative, direct	Tumor tissue (mouse), cell lines	Luciferase assay, RT-qPCR, IHC, WB	[104]
	↑adiponectin, ↑VEGF-C, ↑CAMKII, AMPK, ↑p38	Chondrosarcoma	Correlative, direct	Tumor tissue (mouse), cell lines	Luciferase assay, RT-qPCR, IHC, WB	[129]
	↑VEGF-C	Gastric cancer	Correlative, indirect	Plasma sample (human), cell lines	miRNA microarray, RT-qPCR	[130]
↓miR-300	↑VEGF-C, ↑WISP, ↑ILK, Akt signaling	OSCC	Correlative, direct	Tumor tissue (human), cell lines	Luciferase assay, RT-qPCR, IHC, WB	[131]
↓miR-381	↑VEGF-C, ↑bFGF	Chondrosarcoma	Correlative, direct	Tumor tissue (human), cell lines	Luciferase assay, RT-qPCR, IHC, WB	[132]
↓miR-507	↑VEGF-C, ↑CCL5	Chondrosarcoma	Correlative, direct	Tumor tissue (human, mouse), cell lines	Luciferase assay, RT-qPCR, IHC, WB	[133]
↓miR-624-3p	↑VEGF-C, ↑BDNF, ↑MEK/ERK/mTOR signaling	Chondrosarcoma	Correlative, direct	Tumor tissue (human, mouse), cell lines	Luciferase assay, RT-qPCR, IHC, WB	[134]
↓miR-186	↑VEGF-C, ↑resistin	Chondrosarcoma	Correlative, direct	Tumor tissue (human, mouse), cell lines	Luciferase assay, RT-qPCR, IHC, WB	[103]
↓miR-3178, ↓miR-593-5p, ↓miR-4485, ↓miR-17, ↓miR-469, ↓miR-124-5p	↑VEGF-C (needs further investigation)	Gastric cancer	Correlative, indirect	Tumor tissue (human), cell lines	RT-qPCR, miRNA microarray, Taqman RT-PCR (validation)	[135]
↓miR-126	↑VEGF-A	OSCC	Correlative, direct	Tumor tissue (human), cell lines	Luciferase assay, RT-qPCR, IHC, methylation specification PCR	[136]
↓miR-503-5p	↑VEGF-A, ↑AKT	Colon cancer	Correlative, direct	Tumor tissue (human), cell lines	Luciferase assay, RT-qPCR, WB	[108]
↓miR-221, ↓miR-222	↑ETS1 ↑ETS2	Kaposi’s sarcoma	Direct	Cell lines	Luciferase assay, RT-qPCR, miRNA microarray	[137]
↓circNFIB1	↑miR-486-5p (sponging)/↑VEGF-C	PDAC	Correlative, direct	Tumor tissue (human, mouse), cell lines	Luciferase assay, RT-qPCR, NGS, WB, ISH	[138]
**Pro-lymphangiogenic**						
↑miR-128	↓VEGF-C, ↓ERK, ↓Akt, ↓p38	NSCLC	Correlative, direct	Tumor tissue (human), cell lines	Luciferase assay, RT-qPCR, WB	[127]
	↓VEGF-C	Bladder cancer	Correlative, direct	Tumor, tissue (human, mouse), cell lines	Luciferase assay, RT-qPCR, WB	[128]
↑miR-195-3p	↓VEGF-C, ↓CCL4, ↓JAK2/STAT3 signaling	OSCC	Correlative, direct	Serum (human), tumor tissue (human, mouse), cell lines	Luciferase assay, RT-qPCR, IHC, WB	[139]
↑miR-206	↓VEGF-C, ↓KRAS, ↓NFκB, ↓ANXA2	PDAC	Correlative, direct	Tumor tissue (human, mouse), cell lines	Luciferase assay, RT-qPCR, IHC	[140]
↑miR-182-5p	↓VEGF-C, ↓VEGFA, ↓VEGFR-2, ↓VEGFR-3, ↓ERK, ↓AKT	Colon cancer	Correlative, direct	Tumor tissue (human, mouse), cell lines	Luciferase assay, RT-qPCR, WB	[141]
↑miR-486-5p	↓NRP2	CRC	Correlative, direct	Tumor tissue (human, mouse), cell lines	Luciferase assay, RT-qPCR, IHC, WB	[142]
↑miR-93	↓ANGPT-2	MPE	Correlative, direct	Tissue specimens (human), cell lines	Luciferase assay, RT-qPCR, miRNA microarray	[143]
↑miR-129-5p	↓ZIC2, ↓Hedgehog signaling (Smo, Sli1, Shh)	NPC	Correlative, direct	Tumor tissue (human, mouse), cell lines	Luciferase assay, RT-qPCR, IHC, gene-based microarray, WB	[144]
↑miR-548k	↓ADAMTS1, ↑VEGF-C/VEGFR-3 signaling	ESCC	Correlative direct	Tumor tissue (mouse), cell lines	Luciferase assay, RT-qPCR, IHC, WB	[145]
↑miR-19	↓THBS1, ↑MMP-9/VEGF-C	Colon cancer	Correlative, direct	Tumor tissue (mouse), cell lines	Luciferase assay, functional assays	[146]
↑miR-648, ↑miR-5002-3p, ↑miR-4485, ↑miR-135a, ↑miR-17, ↑miR-1469, ↑miR-124-5p	↑VEGFC (needs further investigation)	Gastric cancer	Correlative	Tumor tissue (human), cell lines	RT-qPCR, miRNA microarray, Taqman RT-PCR (validation)	[135]
↑miR-526b, ↑miR-655	↓PTEN, ↑PI3K/Akt signaling, ↑VEGF family, ↑COX-2, ↑LYVE-1, ↑EP4 (needs further investigation)	Breast cancer	Correlative	Tumor tissue (human), cell lines	RT-qPCR, WB	[147]
↑miR-155	↓BRG1, ↑STAT3/VEGF-C, ↑LYVE-1	NKTCL	Correlative, direct	Tumor tissue (mouse), cell lines	Luciferase assay, RT-qPCR, IHC, WB	[148]
↑miR-221-3p	↓VASH-1, ↑ERK/Akt signaling	CSCC	Correlative, direct	Tumor tissue (human), cell lines	Luciferase assay, RT-qPCR, IHC, ISH, WB	[149]
↑miR-31	↓FAT4	Kaposi’s sarcoma	Direct	Cell lines	Luciferase assay, RT-qPCR, miRNA microarray	[137]
↑miR-27a	↓SMAD4	Colon cancer	Direct	Cell lines	Luciferase assay, RT-qPCR, miRNA microarray, WB	[150]
↑ANRIL	↓CDKN2A, ↑VEGFC, ↑VEGFR-3, ↑LYVE-1	Colon cancer	Correlative, indirect	Tumor tissue (human), cell lines	RT-qPCR, IHC, WB	[90]
↑BLACAT2	↑VEGF-C, ↑WDR5	Bladder cancer	Correlative, direct	Tumor tissue (human, mouse), cell lines	RT-qPCR, NGS, microarray, IHC, ISH, RNA IP, WB, mass spectrometry, ChIRP	[151]
↑HUMT	↑FOXK1, ↑YBX1, ↑Akt/mTOR/VEGF-C signaling	Breast cancer	Correlative, direct	Tumor tissue (human, mouse), cell lines	RT-qPCR, IHC, ISH, FISH, RNA IP, ChIP, WB	[152]
↑ASLNC07322	↓SMAD4, sponging miR-128-3p, ↑VEGF-C	Colon cancer	Correlative, direct	Tumor tissue (human, mouse), cell lines	Luciferase assay, RT-qPCR, ISH, ChIP	[153]
↑HANR	↓miR-296, ↑EAG1/VEGF-A signaling	HCC	Direct	Cell lines	Luciferase assay, RT-qPCR, WB	[154]

Correlative: Correlation of expressions between ncRNAs with samples or tissues applied in the study; direct: Indicates direct binding or interaction between ncRNAs and targeted genes; indirect: no direct interaction or binding between ncRNAs and targeted genes reported in the particular study. ncRNA, non-coding RNA; NSCLC, non-small cell lung cancer; OSCC, oral squamous carcinoma cell; PDAC, pancreatic adenocarcinoma cell; CRC, colorectal cancer; MPE, malignant pleural effusion; NKTCL, natural killer/T cell lymphoma; NPC, nasopharyngeal carcinoma; ESCC, esophageal squamous carcinoma cell; CSCC, cervical squamous carcinoma; HCC, hepatocellular carcinoma; RT-qPCR, real-time quantitative polymerase chain reaction; WB, Western blotting; NGS, next gene sequencing; IHC, immunohistochemistry; ISH, in-situ hybridization; FISH, fluorescence in-situ hybridization; ChIRP, chromatin isolation by RNA purification; ChIP, chromatin immunoprecipitation; RNA IP, RNA immunoprecipitation.

A significant downregulation of miR-27b was found in the plasma of gastric cancer patients. To further elucidate its role, miR-27b was transfected in SGC7901 cells and the results demonstrated that miR-27b transfected cells repressed VEGF-C, induced apoptosis, and inhibited cell proliferation when compared to the negative control. Taken together, these studies show that miR-27b has a direct effect on VEGF-C, and thus abrogates VEGF-C’s function, thereby inhibiting tumor progression [130]. In contrast with miR-27b, miR-27a was reported to be highly expressed in various cancers, including breast, pancreatic, and ovarian cancer [155]. In a study by Xu et al., the overexpression of miR-27a induced the tube lymphatic formation of LECs in a colon cancer microenvironment. miR-27a mediated its effect through binding at the SMAD4 mRNA 3′-UTR region and downregulating the synthesis of the SMAD4 protein, subsequently inducing the lymphangiogenesis of LECs [150].

In oral squamous cancer, lymphangiogenesis was induced by the Wnt-inducible signaling pathway protein-1 or WISP-1, which promotes VEGF-C expression by downregulating miR-300 through integrin signaling αVβ3 and ILK, as well as the Akt signaling pathway [131]. A study by Lien et al. demonstrated an association of miR-195-3p with VEGF-C, mediated by the chemokine CCL4. CCL4 enhanced VEGF-C expression and promoted lymphangiogenesis in oral cancer cells in vitro and in vivo by activation of the JAK2/STAT3 signaling pathway. However, the binding of miR-195-3p inhibited CCL4-induced VEGF-C and exogenous CCL4 reduced the miR-195-3p level [139]. Therefore, it was suggested that miR-195-3p had a direct effect on VEGF-C to suppress lymphangiogenesis.

Exogenous basic FGF (bFGF) has been reported to reduce miR-381 expression and thereby induce VEGF-C, LEC migration, and tube formation in human chondrosarcoma cells. Conversely, the VEGF-C gene also harbors a miR-381 binding site in the 3′-UTR region and an increased expression of miR-381 can negatively regulate VEGF-C-mediated lymphangiogenesis [132]. The downregulation of miR-507 induced VEGF-C-dependent lymphangiogenesis via activation of the chemokine CCL5, as evidenced in a chondrosarcoma xenograft mouse model [133]. A study on pancreatic adenocarcinoma (PDAC) showed a pleotropic effect of miR-206 by modulating multiple cancer targets including VEGF-C, KRAS-induced NF-kB, and the pro-metastatic gene ANXA2, which subsequently reduced cell proliferation, invasion, and tumor lymphangiogenesis [140]. Another finding in human chondrosarcoma showed that miR-624-3p acts as negative regulator for brain-derived neurotrophic factor (BDNF), which facilitates VEGF-C production to induce lymphangiogenesis, migration, and tube formation of LECs cultured with primordial JJ012 cells. Moreover, the mechanistic investigation demonstrated that miR-624-3p was negatively regulated by BDNF-VEGF-C via the MEK/ERK/mTOR signaling pathway [134]. Additionally, the downregulation of miR-186 in human chondrosarcoma tissue was correlated with the upregulation of VEGF-C and the induction of LEC-associated lymphangiogenesis stimulated by a high level of resistin, a cytokine that is secreted from adipocytes and monocytes [103].

The overexpression of miR-548k promotes VEGF-C secretion and stimulates lymphangiogenesis by modulating ADAMTS1/VEGF-C/VEGFR-3 pathways in both in vitro and in vivo models of esophageal squamous cell carcinoma. ADAMTS1 harbors a 3′-UTR complementary site to the seed region of miR-584k, which upon its binding, led to the downregulation of ADAMTS1 [145]. The study revealed that the ectopic expression of ADAMTS1 could reduce the ability of miR-584k on the migration and tube formation of HDLECs. Consistently, increased tube formation was found in HDLECs in conditioned media of ADAMTS1 knockdown cells. ADAMTS1 was reported to form a complex with VEGF-C and attenuate the phosphorylation of VEGFR-3, thus inhibiting lymphangiogenesis [156]. However, the overexpression of miR-548k led to an increased release of VEGF-C from the ADAMTS1 complex, leading to an enhanced induction of lymphangiogenesis in esophageal squamous carcinoma. A more recent study by Yan et al. demonstrated that VEGF-C was post-transcriptionally and negatively regulated by the binding of miR-182-5p to its mRNA 3′-UTR region in colon cancer cells. The overexpression of miR-182-3p clearly suppressed colon cancer cell proliferation, migration, invasion, colony formation, and promoted G1 arrest and apoptosis. In a BALB/c nude mice model, miR-182-3p was associated with a decreased of critical lymphangiogenic and angiogenic factors VEGF-A, VEGFR-2, and VEGFR-3, through the ERK and AKT signaling pathways [141]. miR-19a was reported to be upregulated in CRC cells and a tumor xenograft model. Further mechanistic analysis showed that thrombospondin-1 (THBS-1) was a direct target for miR-19a [146]. LECs treated with a miR-19a inhibitor suppressed their survival, invasiveness, and migration, whereas the matrix metallopepsidase (MMP-9) and VEGF-C were downregulated. THBS-1 silencing attenuated the suppressive effect of the miR-19a inhibitor and also suppressed tumor growth and lymphatic tube formation in vivo through TBHS1-MMP9/VEGF-C signaling [146]. A number of differentially expressed miRNAs were identified between gastric cancer tissue and HLECs co-cultured with VEGF-C-transformed gastric cancer cells. Several miRNAs related to lymphangiogenesis and the lymphatic system were significantly upregulated, including miR-648, miR-5002-3p, miR-4754, miR-4760-5p, miR-4491, miR-4252, miR-5007-3p, and miR-647. In contrast, miR-3178, miR-593-5p, miR-4485, miR-135a-3p, miR-17, miR-1469, and miR-124-5p were downregulated [135]. However, further mechanistic investigation of the miRNAs is warranted as no further validation was performed to confirm the findings.

Recent findings by Hunter et al. demonstrated that the overexpression of miR-526 and miR-655 in the poorly metastatic MCF7 breast cancer cell line resulted in the significant upregulation of lymphangiogenesis and angiogenesis markers, including VEGF-C, VEGF-A, VEGF-D, COX-2, LYVE-1, VEGFR-1, and VEGFR-2. It was hypothesized that the regulation of lymphangiogenesis occurred via two mechanisms: Activation of the prostaglandin receptor (EP4) and the inhibition of PTEN, a tumor suppressor gene that acts as a negative regulator in the PI3K/Akt signaling pathway. It was previously reported that COX-2 is highly expressed in miR-526b and miR-655 breast cancer cells [157,158] and that it can regulate the production of PGE2 and later bind to its receptor, EP4to activate the PI3K/Akt pathway. It was also found that PTEN is a direct target of miR-526b and miR-655 as its downregulation in miR-526b and miR-655 MCF7 cells led to the loss of its inhibitory effect and the upregulation of VEGF expression [147]. However, further investigation is warranted as the exact mechanism of miR-526b and miR-655 in regulating lymphangiogenesis remains unclear. Previous findings have shown that miR-126 is associated with cell growth and acts as a negative regulator of VEGF-A, which plays an important role in lymphangiogenesis, lymph node metastasis, and the recurrence of oral squamous carcinoma cells [136,159]. VEGF-A was also reported to be a negative regulator of miR-503-5p. The downregulation of miR-503-5p resulted in the induction of tumorigenesis, angiogenesis, and lymphangiogenesis in colon cancer by stimulating the AKT signaling pathway [108].

Besides the VEGF family, other lymphangiogenic mediators also harbor a miRNA binding site. NRP-2 was reported to contain a target site for miR-486-5p and the downregulation of miR-486-5p was associated with advanced stage colon cancer [142]. By targeting NRP-2, tumor growth and lymphangiogenesis were significantly suppressed in colorectal carcinoma in a nude mouse model. miR-93 was identified to have an inhibitory effect on malignant pleural effusion by targeting ANGPT2 to regulate angiogenesis and lymphangiogenesis [143].

Computational analysis and further validation in Kaposi’s sarcoma study revealed the involvement of miR-221, miR-222, and miR-31 in endothelial cell motility, through different routes. miR-221 and miR-222 were reported to target tumor progression and hematopoietic development proteins, namely ETS-1 and ETS-2. The downregulation of miR-221 and miR-222, as well as overexpression of ETS1/2, induced the migration of endothelial cells. In contrast, the upregulation of miR-31 enhanced the motility of endothelial cells by repressing proto-cadherin Fat-4 (FAT4), a tumor suppressor in breast cancer that was reported to inhibit tumor growth in [137,160]. Recent findings in cervical squamous cell carcinoma (CSCC) demonstrated the upregulation of miR-221-3p in clinical specimens, which correlated with peritumoral lymphangiogenesis and lymph node metastasis. Transferring CSCC-secreted exosomal miR-221-3p into HLECs significantly induced LEC migration and tube formation when compared to a low-exosomal-miR-221-3p level in cells [149]. The regulation of miR-221-3p was mediated by vasohibin (VASH-1), which later activated the ERK/AKT pathway to induce lymphangiogenesis and lymph node metastasis [149]. Brahma-related-gene 1 or BRG-1 is a catalytic subunit in the SWI/SNF chromatin-remodeling complex and was reported to inhibit the activation of STAT3/VEGF-C signaling, thereby disrupting lymphangiogenesis in colorectal cancer cells [161]. miR-155 was shown to regulate lymphangiogenesis in natural killer/T-cell lymphoma (NKTCL) by targeting BRG-1/STAT3/VEGF-C signaling. Additionally, tumor growth was significantly reduced in an NKTCL xenograft mouse model that was transfected with a miR-155 inhibitor. Interestingly, the upregulation of miR-155 was associated with a decreased level of VEGF-C and LYVE-1, two important markers for LECs and tumor-associated lymphangiogenesis [148]. In a study on nasopharyngeal carcinoma (NPC), miR-129-5p was found to inhibit lymphangiogenesis and lymph node metastasis by blocking the zinc finger ZIC2-mediated Hedgehog signaling pathway. A high expression of ZIC2 was found in NPC tissues when compared to normal tissues. The exogenous expression of miR-129-5p resulted in a reduced expression of ZIC2 and other Hedgehog-signaling components, including Smo, Sli1, Shh, and increased Ptch [144].

A growing number of studies on miRNAs in cell lines, mouse models, non-human primates, and human tissues indicate that they may be promising targets for cancer therapy. Similar approaches to other ncRNAs besides miRNAs are essential as they can provide a deeper understanding on the complexity of lymphangiogenesis-related disease. Moreover, the interplay between ncRNAs and other molecules hinders the translation of ncRNA-based therapy into clinical practice. Therefore, to determine the full extent of mechanisms by which ncRNAs exert their pathological effects, accumulating studies have now started to focus on other ncRNAs, including lncRNAs (Table 1).

#### 4.2.2. lncRNAs and Other ncRNAs in Tumor-Associated Lymphangiogenesis

ANRIL is a lncRNA that can work with polycomb repressive complex-1 (PRC1) and PRC2 to form heterochromatin surrounding the INK4b-ARF-INK4a locus and repress the tumor suppressor genes, such as CDKN2A and CDKN2B [162]. ANRIL overexpression can lead to the induction of cell proliferation and suppression of apoptosis. Additionally, the silencing of CDKN2A by ANRIL can predict lymphovascular invasion and lymph node metastasis in colorectal cancer [163,164]. According to Sun et al., colorectal cancer patients with a high level of ANRIL also expressed a higher level of VEGF-C, VEGFR-3, and LYVE-1, suggesting that ANRIL may act as a driver for lymphangiogenesis in colon cancer. A further investigation showed that the suppression of ANRIL significantly reduced the invasion, migration, and lymphangiogenesis of LECs. Accordingly, mice with downregulated ANRIL had lower tumor growth rates and decreased levels of VEGF-C, VEGFR-3, and LYVE-1. Taken together, these studies show that the downregulation of ANRIL can repress lymphangiogenesis and subsequently inhibit lymphatic metastasis in colon cancer [90].

RNA LINC00958, commonly known as bladder cancer-associated transcript 2 (BLACAT2), was markedly upregulated in human LN-metastatic bladder cancer [151]. BLACAT2 contributed to bladder cancer cell invasiveness, tumor-associated lymphangiogenesis, and lymphatic metastasis in both in vitro and in mouse models. BLACAT2 mediates the regulation of VEGF-C by directly associating with WDR5 (WD repeat containing protein-5), a core unit of the human H3K4 methyltransferase complex that epigenetically regulates target gene expression with H3K4. The overexpression of BLACAT2 dramatically increased WDR5 expression and H3K4 methylation of promoters of VEGF-C, whereas the silencing of BLACAT2 significantly reduced WDR5 expression and H3K4 methylation. Additionally, the same effect of BLACAT2, WDR5, and VEGF-C was also observed in HLECs and SV-HUC-1, a normal bladder epithelial cell line. Furthermore, the administration of anti-VEGF (pV1006R-r) showed promising anti-tumor effects by inhibiting lymphangiogenesis and lymph node metastasis in bladder cancer cells [151].

Another recently discovered lncRNA that is related to tumor lymphangiogenesis is highly upregulated in the metastatic triple-negative breast cancer (HUMT) TNBC-lymph node. A study performed by Zheng et al. revealed that HUMT was significantly higher in the TNBC-cell lines MDA-MB-231 and BT549 compared to non-TNBC cell lines and normal epithelial cells. The total number of branches was significantly decreased and tube length was disrupted in the HUMT-knock out HLECs compared to the normal cells, suggesting that HUMT promotes proliferation, lymphangiogenesis, and lymph node metastasis in TNBC in vitro. Furthermore, the HUMT expression was markedly increased in lymph node-invasive cells due to DNA hypomethylation at the promoter region of forkhead-box K1 (FOXK1). A strong correlation was found between HUMT, FOXK1, and Y-box binding protein 1 (YBX1). HUMT exhibited its function by recruiting YBX1, a well-known RNA/DNA binding protein, and formed a transcription complex to enhance the expression of FOXK1, and eventually leading to the downstream alteration and activation of the lymph node metastatic-related Akt/mTOR/VEGF-C pathway [152].

Numerous studies have reported the function of SMAD4 as a negative regulator of VEGF-C in various types of cancer. SMAD4 negatively regulates VEGF-C through the increase of miR-128-3p, by promoting the nuclear entry of phosphorylated Smad3 that binds to the promoter of miR-128-3p to promotes its transcription before binding to the 3′-UTR of the VEGF-C gene. The presence of the lncRNA ASLNC07322 has been reported to reduce miR-128-3p in metastatic colon cancer and ASLNC07322 knockdown inhibited the cell proliferation and lymphangiogenesis of LECs. Furthermore, combined ASLNC07322 knockdown with the overexpression of SMAD4 had a synergistic effect on the inhibition of lymphangiogenesis and proliferation, as well as increased apoptosis induction of LECs and tumor growth in a mouse xenograft model. Mechanistically, the inhibitory effect of miR-128-3p on VEGFC has been shown to be inhibited by ASLNC07322, which acts as a miRNA sponge for miR-128-3p, leading to the subsequent elevation of VEGFC [153,165].

HCC-associated long non-coding RNA or HANR, was identified as an oncogenic factor and was shown to induce proliferation and chemoresistance in hepatocellular carcinoma (HCC) [166,167]. In a recent study, a high expression of HANR was reported to induce tumor lymphangiogenesis in the HCC microenvironment. HANR directly interacted with miR-296 and induced the secretion of exosomal miR-296 from HCC cells to modulate the lymphangiogenesis of LECs. The mechanistic study showed that the knockdown of HANR reduced VEGF-A and the potassium channel family EAG1, which is known to enhance cell proliferation in a number of cancer cell lines [168,169]. Interestingly, LECs treated with exosomes containing miR-296 showed lower expression levels of VEGF-A when co-incubated with conditioned medium of HANR-silenced HCC cells [154].

In a recent study, circRNA (circNFIB1, has-circ_0086375) was found to be downregulated in pancreatic cancer and negatively associated with lymph node metastasis in PDAC patients. The mechanistic investigation showed that circNFIB1 acted as a sponge and diminished the oncogenic effect of miR-486-5p to induce PI3KR1 expression, thereby downregulating VEGF-C, which subsequently suppressed lymphangiogenesis and lymph node metastasis in PDAC cells [138]. In contrast with miRNAs, all lncRNAs reported have been proven to play a pro-lymphangiogenic role in tumor-associated lymphangiogenesis, but further functional studies are needed to verify their other roles (Figure 3B).

## 5. Conclusions

Lymphangiogenesis is a key event in cancer metastasis that could be targeted to prevent or reduce metastatic progression. Therefore, the identification of ncRNAs regulating this process could lead to the development of targeted anti-metastatic cancer therapies. While anti-angiogenesis research is continuously accelerating, the growing interest in anti-lymphangiogenic drugs has not yet been fully translated from the bench to bedside. Despite the increasing numbers of ncRNAs as potential drug targets, there is much research that still needs to be done. Targeting tumor lymphatics as a cancer therapy is complicated, considering that the lymphatic system is unidirectional, so inhibiting the function of vessels could hamper the lymphatic drainage. Therefore, the development of anti-lymphangiogenic therapies that are based on ncRNAs would be a promising alternative to prevent cancer metastasis, but it is also crucial to preserve the normal physiology of the lymphatic system. As shown in this review, ncRNAs play significant roles in regulating lymphatic vasculature in normal and pathological conditions. Moving forward, extensive investigations into the mechanisms by which ncRNAs regulate lymphangiogenesis and large-scale studies are required before ncRNA-based therapy for tumor lymphangiogenesis can be moved into the clinical setting.
